# Medical Opinion and Movement

**Published:** 1907-06-01

**Authors:** 


### Medical Opinion and Movement.

An interesting discussion took place at a meeting of the North of England Obstetrical and Gynæcological Society on the treatment of eclampsia. It was generally agreed that no great advances had been made in our knowledge of the disease and its treatment. More rational methods of treatment have, however, undoubtedly followed the view that it is a toxæmic condition, and that measures should be directed towards the elimination of the poison. Such means consist in promoting diaphoresis, saline infusions, saline rectal injections, and lavage. The convulsions should be controlled by morphine and chloroform, and the onset of labour hastened. There was a consensus of opinion against *accouchement forcé*. Rupture of the membranes, dilatation of the cervix, acceleration of delivery, and forceps rather than version, were generally approved. Dr. Stookes showed from statistics that there has been a considerable increase of the disease during the last ten years, and he ascribed this to the increase of intestinal affections.

It frequently happens in the surgical wards of the hospitals that patients have to be detained during convalescence for a prolonged period on account of some necessary surgical care, although possibly of a trivial nature, or owing to an urgent demand for beds a patient may have to leave the hospital while some such care is still needful. In order to provide for these cases it has been proposed to establish, in connection with the London Hospitals a surgical home of recovery in which the surgical treatment of convalescents can be effectively carried on. A meeting was held this month at the Mansion House by Alderman Sir Walter Vaughan Morgan, in aid of the movement, and representatives from most of the London hospitals were present, as well as Mr. Mayo Robson, Mr. Stephen Paget, and Sir Alfred Fripp. The home has been incorporated under a trust, with Her Royal Highness Princess Louise, Duchess of Argyll as President. An appeal is now being made for the sum of £30,000 to build and start the home, and it is to be hoped that the movement will receive the support it deserves.

There will probably always be some differences of opinion in regard to the question of operative interference in appendicular conditions. At the present time there is no doubt that a large number of appendices are removed, which might quite well have been left alone, and, on the other hand, lives are still lost through delay in operating. Signs and symptoms are so often misleading that even the elect cannot fail to be deceived sometimes, and the opinion of an individual surgeon must vary from time to time according as his immediate experience has tended in the one direction or the other. At present there is a tendency among French medical authorities towards the more conservative treatment, and Dr. Saint René Bonnet of Chatel-Guyon-les-Bains champions this view, and especially deprecates removal of the appendix in cases of muco-membranous entero-colitis with appendicular symptoms. He claims that these cases yield to medical treatment, and that the entero-colitis is not cured by removal of the appendix. On the other hand, M. Richelot maintains that such intractable intestinal complaints are often cured by the removal of an appendix, and he supports his view by many cases which have been successfully treated in this way.

The bacterial origin of acute rheumatism cannot be said to be yet definitely determined. Several observers have from time to time succeeded in isolating a streptococcus from cases of the disease, and five or six years ago, owing chiefly to the researches of Poynton, Paine, Walker, Beaton and Ryffel, there seemed good prospects that the question would be speedily settled and the specific organism clearly identified and labelled. In a recent paper Dr. E. W. A. Walker, who by his researches has made the subject peculiarly his own, reviews the whole question from a thoroughly scientific and unbiased standpoint. Poynton and Paine succeeded in isolating a streptococcus in some 30 cases of acute rheumatism, and obtained all the manifestations of the disease by inoculations in animals. Walker and Beaton isolated a similar organism from 15 cases, and they obtained similar results to Poynton and Paine on injecting cultures of the organism into rabbits. Fever, acute polyarthritis, endocarditis, and pericarditis were manifested.

Identical results were also obtained by Dr. Vernon Shaw, working with the same cultures on rabbits and monkeys. Considerable difficulty has been encountered in the attempt to establish distinguishing features between this so-called micrococcus rheumaticus and other streptococci. Walker and Ryffel were able to show, however, that when this organism is grown in highly alkaline media, a considerable amount of formic and acetic acid are produced, and they determined the amount in relation to the alkalinity of the medium. On the other hand only very small amounts of formic acid are produced by other strains of streptococci. Incidental to this research these observers examined the urine of patients suffering from rheumatic fever, and found abnormal quantities of formic acid present, amounting to as much as 0.25 gram in a day's urine, while, according to Von Jaksch, the total amount of fatty acids in normal urine is no more than 0.05 gram per day. It was found moreover that the effect of treatment with salicylates was to cause a rapid fall in the formic acid excretion. All these facts seem to harmonise very prettily. But recently Bulloch and Thompson have carefully investigated the subject anew, and examining 14 cases during life and 20 cases after death, they have failed to discover any organism. Cole, Philip and others have met with similar results. From clinical and pathological aspects there can be little or no doubt that the disease is due to an infective micro-organism, probably of an "attenuated" nature, but the identification of the organism must still remain *sub judice*.

